# The Role of the Intraoperative Optical Coherence Tomography for Vitreoretinal Surgery in a Real-Life Setting

**DOI:** 10.3390/life13091813

**Published:** 2023-08-27

**Authors:** Barbara Parolini, Matteo Ripa, Rino Frisina, Veronika Matello, Lorenzo Motta

**Affiliations:** 1 Department of Ophthalmology, Eyecare Clinic, 25124 Brescia, Italyveronika.matello@gmail.com (V.M.); 2 Department of Ophthalmology, William Harvey Hospital, East Kent Hospitals University NHS Foundation Trust, Ashford TN24 0LZ, UK; 3 Ophthalmology Unit of Surgery, Department of Guglielmo da Saliceto Hospital, 29121 Piacenza, Italy

**Keywords:** OCT, intraoperative OCT, i-OCT, retina, surgery, vitreoretinal surgery

## Abstract

Background: To descriptively report the advantages and the feasibility of microscope-integrated intraoperative optical coherence tomography (i-OCT) in managing different vitreoretinal diseases in a real-life setting. Methods: We conducted an observational retrospective study involving 265 eyes that underwent elective retinal surgery and intraoperative OCT between 1 September 2018 and 1 October 2022 at Eyecare Clinic (Brescia, Italy). Results: 52 epiretinal membranes, 30 retinal detachments, 60 high myopic eyes, 30 choroidal transplants, 40 macular holes, and 32 vitreo-proliferative retinopathies underwent vitreoretinal surgery and intraoperative OCT scans. The i-OCT was a useful diagnostic exam for all cases and significantly influenced our surgical management. Conclusions: i-OCT is a helpful surgical tool in ophthalmic surgery as it provides real-time feedback of tissue anatomy to surgeons, thereby guiding decision-making. Moreover, it provides additional information on the microarchitectural changes after instrument–tissue interactions, further guiding procedures when necessary and possibly reducing unessential surgical maneuvers.

## 1. Introduction

Optical coherence tomography (OCT) is a noninvasive imaging modality based on low-coherence interferometry that provides an in vivo cross-sectional view of biological tissues due to their property of reflecting and backscattering light [1,2,3]. Nowadays, it is used in several specialties such as ophthalmology, cardiology, otology, and dermatology [4,5]. Indeed, this technology offers elements that are useful to reaching a preoperative diagnosis and the postoperative follow-up of different diseases [6].

Intraoperative optical coherence tomography (i-OCT) refers to the application of OCT that allows the surgeon to display real-time tomographic images of the tissues during surgery.

Initially, the most common systems used for i-OCT were portable OCT probes that were either mounted to a microscope or used in a handheld fashion. Prototypes were also integrated into the microscope to allow for the true real-time imaging of instrument–tissue interactions. Nowadays, i-OCT has been fully embedded into the surgical ophthalmic microscope and has demonstrated utility in anterior segment surgery, including corneal transplants and intraocular lense implantation, and in vitreoretinal surgery, including epiretinal membrane (ERM) peeling perfluorocarbon liquid (PFCL) removal and subretinal injections [7,8,9,10].

In 2005, i-OCT was first adopted by surgeons to view anterior segment structures during anterior segment procedures such as deep anterior lamellar keratoplasty and trabeculectomy [11]. In 2014, the Prospective Intraoperative and Perioperative Ophthalmic Imaging with Optical Coherence Tomography (PIONEER) study evaluated the feasibility of this tool in both anterior and posterior segment surgical procedures to establish the utility of i-OCT in the surgical management of ophthalmic disease. This was the first large prospective study which reported the use of a portable intraoperative OCT for anterior (275 cases) and posterior segment (256 cases) surgery, where surgeons reported to have been influenced by intraoperative OCT in 8% of cases [12].

Three OCT prototypes, including the Rescan 700, the EnFocus, and a research prototype, were utilized in the DISCOVER study to assess microscope-integrated i-OCT. A total of 820 patients were enrolled for anterior or posterior segment surgeries over three years. The DISCOVER trial provided additional evidence for the clinical usefulness of i-OCT. Indeed, the i-OCT provided useful information in 29.2% of posterior segment surgeries [13]. Afterward, several studies corroborated the results found in the previous clinical trials, especially for vitreoretinal surgery [14,15].

Currently, three systems are approved by the US Food and Drug Administration. The Zeiss Rescan 700 was the first FDA-approved microscope-integrated i-OCT system that was built on the Lumera 700 (Carl Zeiss Meditec AG, Jena, Germany) microscope platform, which allows the surgeon to visualize i-OCT data without the use of an external monitor, providing immediate feedback to surgeons through the microscope oculars without needing to look away from the surgical field. Moreover, it is also integrated into the microscope foot-pedal with surgeon-directed control of aiming, orientation, and the size of the OCT scan pattern [16].

The Haag-Streit i-OCT system (Haag-Streit, Koeniz, Switzerland) uses the OPMedT (OPMedT, Lubeck, Germany) OCT system and includes a microscope-mounted viewing screen and a heads-up display screen [15]. Finally, the EnFocus (Leica/Bioptigen, Durham, NC, USA) is another microscope-integrated i-OCT option for the Leica surgical microscope that provides an increased range of real-time information for a deeper understanding of how subsurface tissue is reacting to surgical maneuvers [17].

Our study aimed to descriptively report the advantages and the feasibility of i-OCT in managing different vitreoretinal diseases in a real-life setting according to our experience. Furthermore, we also evaluated the potential drawbacks of the i-OCT in a large sample size of patients who underwent elective vitreoretinal surgery in our center.

## 2. Materials and Methods

### 2.1. Patients

In this observational retrospective study, we collected data from 265 eyes that underwent elective vitreoretinal surgery with a microscope-integrated i-OCT between 1 September 2018 and 1 October 2022 at Eyecare Clinic (Brescia, Italy). This study followed the tenets of the Declaration of Helsinki for research involving human subjects. Informed consent was obtained from all subjects to use the data for the study. We included all patients, regardless of age, who were able to provide informed consent, and who were undergoing elective vitreoretinal surgery and i-OCT for posterior segment disease. For patients under 18, written consent signed by both parents was required. All types of vitreoretinal surgeries were included.

All of the surgical cases were operated by one expert surgeon (B.P.) that used the Leica Proveo 8 surgical microscope and the Leica Enfocus ultra HD i-OCT system. This i-OCT has a wavelength of 860 nm with a 90 nm bandwidth.

All of the diagnoses and advised surgical procedures were recorded. Patients whose i-OCT images were not satisfactory according to a prespecified protocol were excluded.

### 2.2. Preoperative and Intraoperative Examination

All patients underwent routine ophthalmologic examinations, including the measurement of best-corrected distance visual acuity (BCVA) in the logarithm of the minimum angle of resolution (logMAR), the measurement of intraocular pressure (IOP) with a non-contact tonometer, a slit lamp bio-microscopy, and a dilated fundus evaluation using a 90 diopters (D) lens. Afterward, a single trained technician (VM) took all retinal images comprised of widefield color fundus retinography and standard macular and peripheral OCT.

During the elective vitreoretinal surgery, the i-OCT was used by the same technician (VM). This OCT provided real-time OCT imaging during surgery and immediate feedback to the surgeon. i-OCT images were detected by the surgeon and the technician during the whole surgical procedure. The most important surgical steps were fully recorded, selecting single screenshots. Imaging data were reviewed during surgery and harvested postoperatively for further analysis.

Continuous i-OCT image acquisition during the surgical maneuver was referred to as real-time image acquisition, whereas image acquisition before and/or after the maneuver was defined as Static i-OCT image acquisition.

### 2.3. Intraoperative Images Acquisition

According to the specific case, different surgical techniques were used, such as scleral or macular buckling, and pars plana vitrectomy performed with either 23- or 25-gauge instrumentation using Stellaris Elite (Bausch & Lomb, Rochester, NY, USA). Retinal imaging was performed either through a disposable flat contact lens (field of view: 36°, magnification 1.02×) or a non-contact wide-field viewing system (BIOM f = 200 mm) according to the specific surgical case. Images were captured in the form of videos for different surgical steps.

For the recording of each video, the technician activated the automatic image optimization. The Auto Locate function was used to center the OCT image automatically, and the location lock in the z-direction further ensured that the image remained centered throughout the procedure. Whenever this procedure was not possible, every video was optimized manually by calibrating the scan range and focus. The surgeon was able to reproduce the same procedure by using the footswitch.

Intraoperatively, we used a variable width of scan ranging from 3 to 20 mm with a scan density of 1,000,000 A-scans max. The standard scan rate was 100,000 A-scans in 3 s. The depth of the scan was 2.5 mm. The axial resolution was ≤4 μm.

During each procedure, the surgeon’s feedback was documented using a standardized case report form, which included comments on the surgical step, instruments used, and image quality. In the postoperative notes, surgical workflow, and the ability to confirm the precision of the maneuver, to offer new information, and to highlight the hole compared to dye staining in high myopic eyes were also documented.

### 2.4. Images Selection and Evaluation

After surgery, two OCT specialists (B.P. and L.M.) examined all intraoperative images according to a pre-specified protocol for quality assessment. If consensus could not be reached, a third specialist (M.R.) was consulted for the final decision. The protocol evaluated the image quality by using five parameters: motion (image interruptions), decentration (center of scan not aligned with the center of the macula), defocus (reduced retinal reflectivity due to poor autofocus), masking (light blockage due to anterior or posterior ocular abnormalities such as vitreous opacities and pigment clumps, which do not permit the beam to reach deeper layers), and segmentation errors (unclear boundary of the different layers of the retina) as previously published [18].

Outside of the protocol, the surgeon (B.P) evaluated to what extent and in which aspects the use of the i-OCT influenced the decision-making process during surgery. Finally, the images were discarded if at least one artifact was identified.

### 2.5. Statistical Analysis

As per the protocol, the interobserver agreement was calculated using a kappa (κ) statistic [19]. The κ statistics were calculated and assessed as follows: <0.20, poor; 0.21–0.40, fair; 0.41–0.60, moderate; 0.61–0.80, substantial; and 0.81–1.00, almost perfect agreement. Data collected included indication of surgery, type of procedure, ocular comorbidities, and specific surgical maneuvers performed.

The statistical analysis was performed using Statistical Package for Social Sciences (SPSS version 27.0, IBM Statistics, Chicago, IL, USA). Numerical variables were calculated as mean ± standard deviation (SD), whereas descriptive variables were calculated in frequencies and percentages. We did not run any inference statistical analysis.

Outside of the protocol, the surgeon offered a qualitative judgment on the images.

## 3. Results

We examined 265 eyes of 265 patients. The study population consisted of 131 males (49.43%) and 134 females (50.57%), with a mean age of 58 years (range: 10–87 years). The demographics and diagnosis features are listed in Table 1 and Table 2. The interobserver agreement between the two examiners was not inferior to 0.93 for each comparison.

### 3.1. Epiretinal Membrane (ERM)

Fifty-two (19.62%) eyes diagnosed with primary ERMs were operated on.

**Figure 1:**
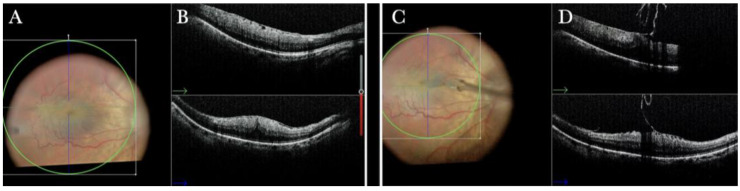
(**A**) Microscope view of the macular area with an epiretinal membrane (ERM) after staining with Doubledyne blue. (The white box indicates the field-of-view of the OCT scan, the green circle within the white box represents the effective working distance and green and blue lines within the green circle show the vertical and horizontal scan planes). (**B**) The intraoperative optical coherence tomography (i-OCT) horizontal (green arrow) and vertical (blue arrow) scan showing the presence of the ERM and the macular thickening. (**C**) Microscope view of the macular area during peeling. The ERM is elevated. (The white box indicates the field-of-view of the OCT scan, the green circle within the white box represents the effective working distance and green and blue lines within the green circle show the vertical and horizontal scan planes). (**D**) The i-OCT B horizontal (green arrow) and vertical (blue arrow) scan during peeling. The ERM is elevated, and the macular profile is preserved where the ERM has been peeled.

**Figure 2:**
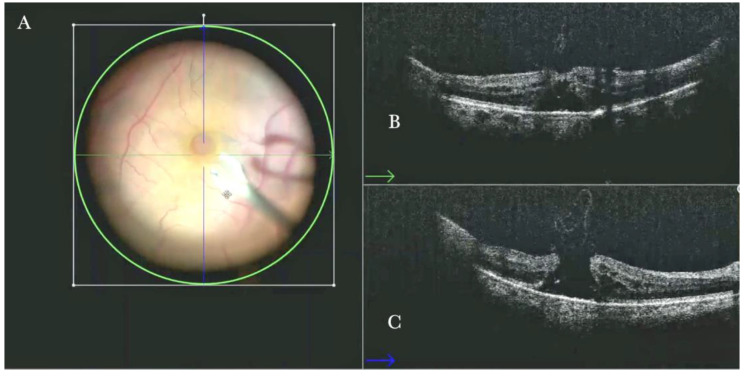
(**A**) Microscope view of the macular area with a macular hole after staining and peeling of the inner limiting membrane (ILM) with the creation of an ILM flap. (The white box indicates the field-of-view of the OCT scan, the green circle within the white box represents the effective working distance and green and blue lines within the green circle show the vertical and horizontal scan planes). (**B**) Intraoperative optical coherence tomography (i-OCT) B horizontal (green arrow) scan showing the ILM flap over the hole. (**C**) i-OCT B vertical (blue arrow) scan showing the ILM flap over the hole.

**Figure 3:**
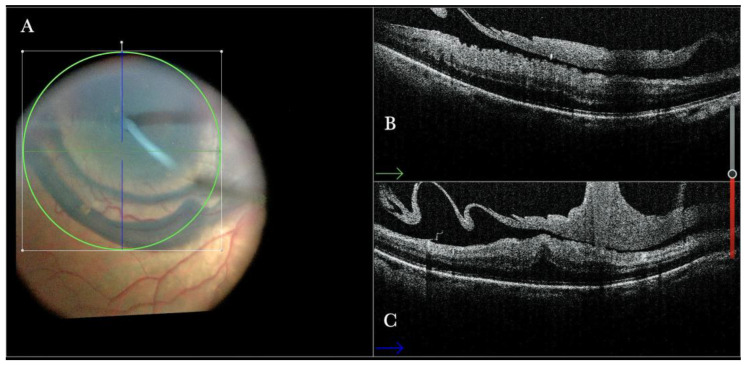
(**A**) Microscope view of the macular area with an epiretinal membrane (ERM) after staining with Doubledyne blue. (The white box indicates the field-of-view of the OCT scan, the green circle within the white box represents the effective working distance and green and blue lines within the green circle show the vertical and horizontal scan planes). (**B**) Intraoperative optical coherence tomography (i-OCT) B horizontal (green arrow) scan showing the macular profile after peeling and restaining with Doubledyne blue. (**C**) i-OCT B vertical (blue arrow) scan showing the macular profile after peeling and restaining with Doubledyne blue.

**Figure 4:**
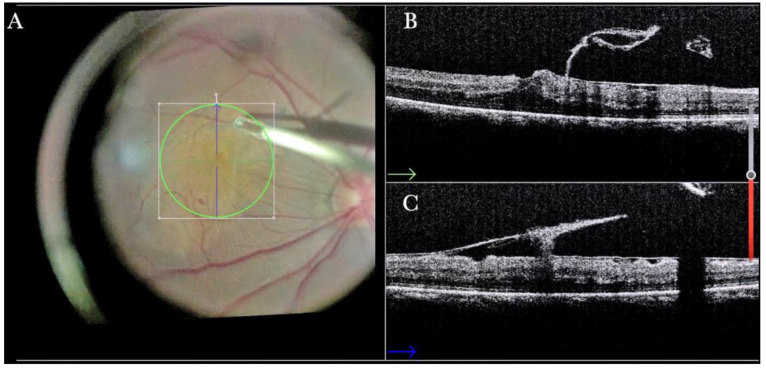
(**A**) Microscope view of the macular area during peeling of an epiretinal membrane (ERM). The ERM is elevated. (The white box indicates the field-of-view of the OCT scan, the green circle within the white box represents the effective working distance and green and blue lines within the green circle show the vertical and horizontal scan planes). (**B**) Intraoperative optical coherence tomography (i-OCT) B horizontal (green arrow) scan during peeling. The ERM is elevated, and the macular profile is preserved where the ERM has been peeled. (**C**) i-OCT B vertical (blue arrow) scan during peeling. The ERM is elevated. The inner limiting membrane (ILM) is stretched and elevated where the ERM is peeled. The i-OCT allowed us to see the double simultaneous peeling of ERM and ILM.

**Figure 5:**
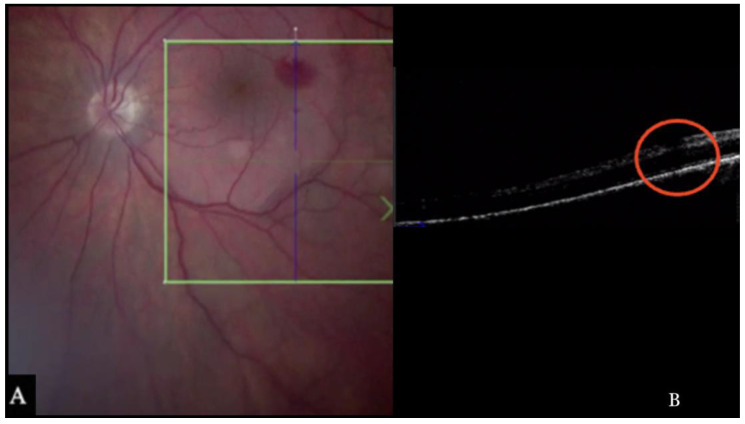
(**A**). Microscope view of the macular area during peeling of an epiretinal membrane (ERM). An intraretinal hemorrhage is present. (The green box indicates the field-of-view of the OCT scan, the green and blue lines within the green box show the vertical and horizontal scan planes). (**B**) Intraoperative optical coherence tomography (i-OCT) B vertical scan showing that the retina is preserved under the hemorrhage. (Red empty circle).

In primary ERMs, the i-OCT was as satisfactory as the dye staining in visualizing both the ERM (Figure 1) and the ILM (Figure 2), and enhancing well the visualization of the dynamic of the staining (Figure 3) and the membranes with architectural changes after the peel. The i-OCT was visualized when the ERM and the ILM were peeled simultaneously (Figure 4).

i-OCT was useful in excluding iatrogenic hole induction by the peeling maneuvers. Indeed, during peeling, bleeding might occur due to the pull maneuvers on the tissue. One of the main strengths of i-OCT was excluding an iatrogenic retinal break under bleeding and under the difficult to achieve view of the air tamponade, as shown in Figure 5.

Supplementary Material Video S1 shows the role of the i-OCT as a useful additional tool in the epiretinal membrane (ERM) flap grasping maneuver.

Supplementary Material Video S2 shows the role of the i-OCT as a useful additional tool in the difficult gliotic epiretinal membrane (ERM) flap grasping maneuver for a colleague in training.

Supplementary Material Video S3 shows a posterior vitreous detachment enhanced visualization with Doubledyne staining and confirmation that no retinal breaks have been induced.

In Supplementary Material Video S4, the intraoperative optical coherence tomography (i-OCT) shows that no iatrogenic macular hole was induced during peeling.

### 3.2. Primary and Recurrent Macular Holes

**Figure 6:**
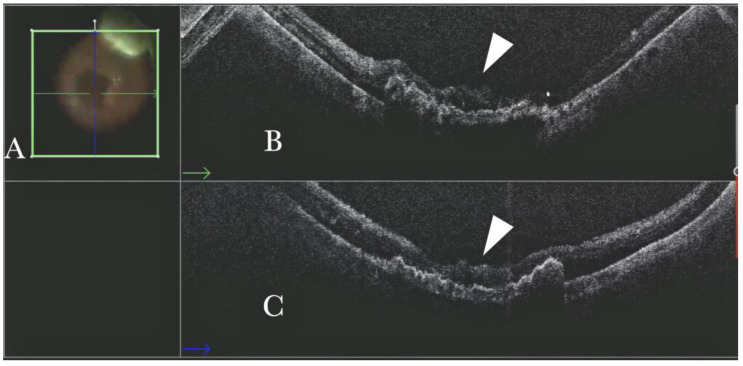
(**A**) Microscope view showing the macular area after autologous choroidal transplant. A full-thickness patch of the choroid has been transplanted from the peripheral area under the macula. A macular hole occurred. (The green box indicates the field-of-view of the OCT scan, the green and blue lines within the green box show the vertical and horizontal scan planes). (**B**) The intraoperative optical coherence tomography (i-OCT) B horizontal (green arrow) scan shows the retina attached over the choroidal patch. An autologous patch of the retina (white arrowhead) was transplanted into a macular hole. (**C**) The i-OCT B vertical (blue arrow) scan shows the retina attached over the choroidal patch. An autologous patch of the retina (white arrowhead) was transplanted into a macular hole.

Forty (15.09%) eyes diagnosed with either primary or recurrent macular holes (MHs) were treated. In primary MHs, the i-OCT could confirm the correct positioning of the ILM flap on the hole (Figure 2) in simple cases and in more difficult cases. A complex case of persistent macular hole is presented in Figure 6 and Supplementary Video S5, which shows hole closure by autologous retina transplant (ART).

Another case of persistent MH is presented in Supplementary Material Video S6. The ex-vacuum technique was applied by injecting silicone oil 1000 and aspirating fluid at the level of the hole with a 41-gauge needle and a backflush needle. The i-OCT showed no change in the hole size and shape. The surgeon also tried to mobilize the edges with a scraper, but the edges remained fixed in the original position. Therefore, a decision to stuff the hole with a free ILM flap was made, leading to hole closure. This interesting case shows how i-OCT guides the choice of technique for macular hole closure.

**Figure 7:**
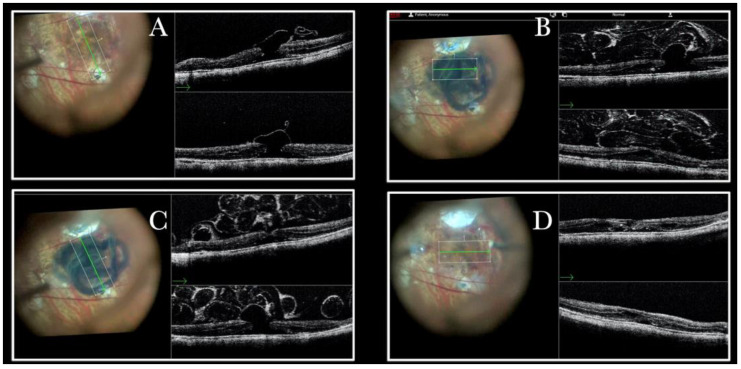
(**A**) Microscope view, and horizontal (green arrow) and vertical view, of a case of a macular hole where an inner limiting membrane (ILM) flap has been created in a case of myopic macular hole. (The white box indicates the field-of-view of the OCT scan, the green line within the green box shows the scan plane). (**B**) Same case, where viscoelastic stained with Doubledyne blue has been injected over the ILM flap to prevent dislocation. The intraoperative optical coherence tomography (i-OCT) shows that the ILM flap remains elevated. (The white box indicates the field-of-view of the OCT scan, the green line within the green box shows the horizontal scan plane). (**C**) Same description as in B with a B (green arrow) scan. (The white box indicates the field-of-view of the OCT scan, the green line within the green box shows the scan plane). (**D**) The viscoelastic material has been replaced by perfluorocarbon liquid (PFCL). The i-OCT scan shows that, under PFCL, the ILM flap is well allocated over and into the hole. (The white box indicates the field-of-view of the OCT scan, the green line within the green box shows the scan plane).

In Figure 7 and in Supplementary Material Video S7, we observe the ILM flap positioned over the hole under the fluid tamponade. To avoid ILM dislocation during the fluid–air exchange (FAEx), the surgeon applied viscoelastic material over the hole that was stained with Doubledyne (Bausch & Lomb). Observing that the ILM flap was not pushed over the hole with viscoelastic material, the surgeon substituted the viscoelastic material with PFCL. The hole then appeared closed with an ILM flap that was strictly positioned over the hole, and a final FAEx could allow us to perform the case.

### 3.3. High Myopia and Myopic Traction Maculopathy (MTM)

**Figure 8:**
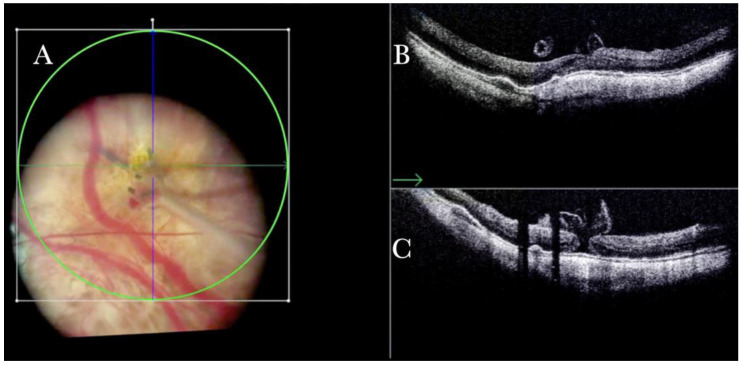
(**A**) Microscope view of the macular area with a macular hole in case of high myopia after staining and peeling of the inner limiting membrane (ILM) with the creation of an ILM flap under fluid. The macular hole is very difficult to highlight due to low contrast in the choroid and confounding details from the choroidal vessels and microhemorrhages. (The white box indicates the field-of-view of the OCT scan, the green circle within the white box represents the effective working distance and green and blue lines within the green circle show the vertical and horizontal scan planes). (**B**) The intraoperative optical coherence tomography (i-OCT) B horizontal (green arrow) scan shows, very clearly, the ILM flap well-positioned over the hole. (**C**) i-OCT B vertical scan showing, very clearly, the ILM flap well-positioned over the hole.

**Figure 9:**
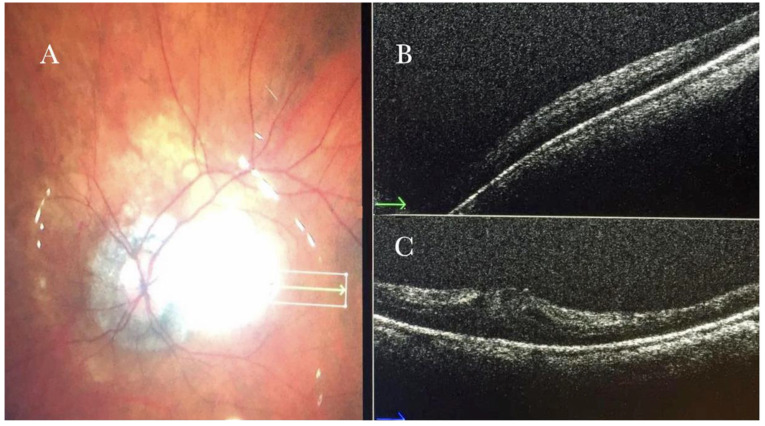
(**A**) Staining and peeling of the inner limiting membrane (ILM) with the creation of an ILM flap under air after fluid air exchange. The macular hole is very difficult to highlight due to low contrast in the choroid and confounding details from the choroidal vessels and microhemorrhages. (The white box indicates the field-of-view of the OCT scan, the green line within the green box shows the scan plane) (**B**) The intraoperative optical coherence tomography (i-OCT) B horizontal (green arrow) scan shows, very clearly, the ILM flap well-positioned over the hole under the air. (**C**) i-OCT B vertical (blue arrow) scan shows, very clearly, the ILM flap well-positioned over the hole under the air.

Sixty (22.64%) high myopic eyes were treated. In these eyes, it is common to find atrophic areas at the posterior pole, and MH repair and ERM peeling are challenging due to the poor contrast between the retina and the choroid. i-OCT was a useful aid in assisting with peeling maneuvers and visualizing the hole edges. Moreover, it represented a great asset in helping the surgeon to correctly place the ILM flap in the right position during the MH repair in high myopic eyes when working under fluid (Figure 8 and Supplementary Material Video S8) and under air (Figure 9).

**Figure 10:**
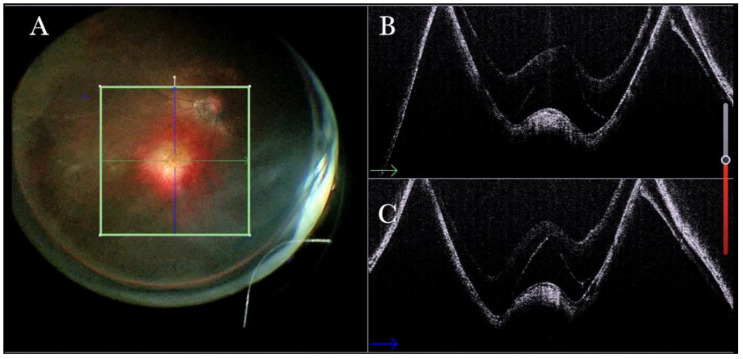
(**A**) Microscope view of the macular area in case of myopic traction maculopathy in stage 3a (macular detachment and no macular hole) after implanting a macular buckle. The transillumination of a fiber optic inserted into the buckle allows us to identify the location of the buckle itself. (The green box indicates the field-of-view of the OCT scan, the green and blue lines within the green box show the vertical and horizontal scan planes). (**B**) The intraoperative optical coherence tomography (i-OCT) B horizontal (green arrow) scan shows, very clearly, the indentation of the macula from the scleral side due to the presence of a macular buckle. (**C**) The i-OCT B vertical (blue arrow) scan shows, very clearly, the indentation of the macula from the scleral side due to the presence of a macular buckle.

**Figure 11:**
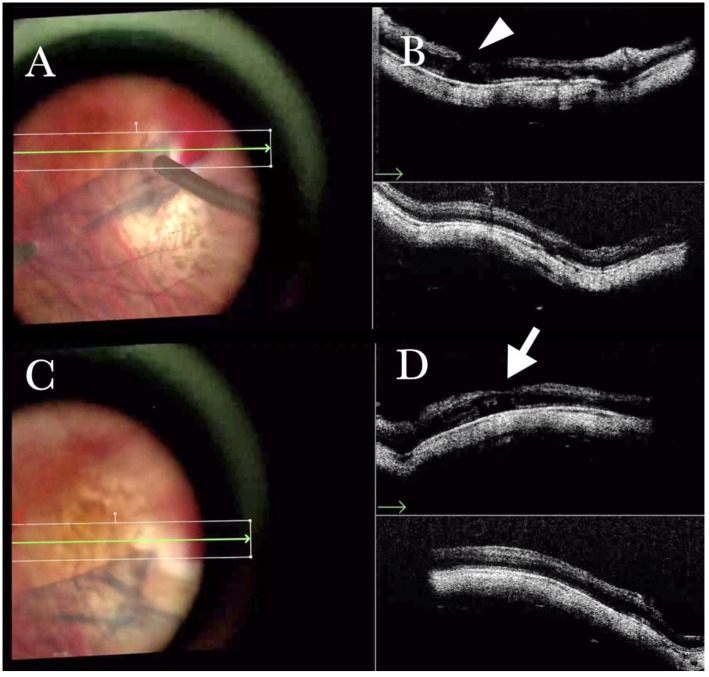
(**A**) Microscope view of the macular area in case of myopic traction maculopathy in stage 4C (macular detachment with full-thickness macular hole (FTMH)) after implanting a macular buckle. The transillumination of a fiber optic inserted into the buckle allows us to guess the location of the buckle itself. The inner limiting membrane (ILM) flap is visibly stained blue. The FTMH is not visible. (The white box indicates the field-of-view of the OCT scan, the green line within the green box shows the scan plane) (**B**) The Intraoperative Optical coherence tomography (i-OCT) B horizontal (green arrow) and vertical scan shows very clearly the indentation of the macula from the scleral side due to the presence of a macular buckle. The white arrowhead shows the FTMH, which is not over the buckle. (**C**) Same view as in A after repositioning the macular buckle. (The white box indicates the field-of-view of the OCT scan, the green line within the green box shows the scan plane) (**D**) i-OCT B horizontal (green arrow) and vertical scan showing very clearly the indentation of the macula from the scleral side due to the presence of a macular buckle. The white arrow shows the FTMH, which is now well-positioned over the buckle.

**Figure 12:**
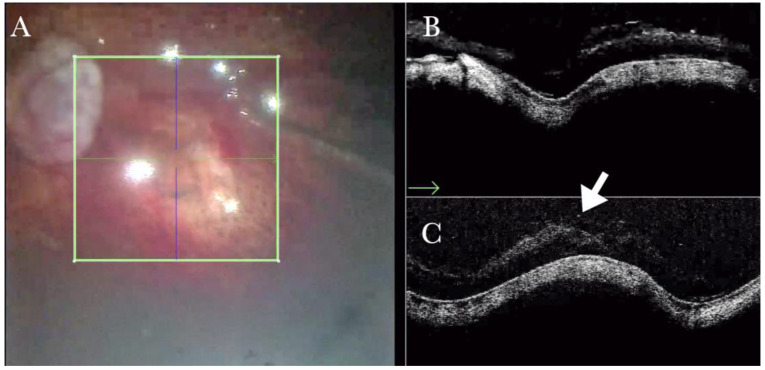
(**A**) Microscope view of the macular area in case of myopic traction maculopathy in stage 4C (macular detachment with full-thickness macular hole (FTMH)) after implanting a macular buckle and peeling the inner limiting membrane (ILM) creating an ILM flap. The transillumination of a fiber optic inserted into the buckle allows us to guess the location of the buckle itself. The FTMH is not visible under the air. (The green box indicates the field-of-view of the OCT scan, the green and blue lines within the green box show the vertical and horizontal scan planes). (**B**) The intraoperative optical coherence tomography (i-OCT) B horizontal (green arrow) scan shows, very clearly, the indentation of the macula from the scleral side due to the presence of a macular buckle. (**C**) The i-OCT B vertical scan shows, very clearly, the indentation of the macula. The white arrow shows the FTMH, which is now well-positioned over the buckle, and the presence of the ILM flap even under the air.

MTM in any stage, according to the MTM staging system (MSS), is one of the most challenging diseases. Among the different surgical options for MTM, the macular buckle represents a satisfactory option that can be used alone to treat cases of maculoschisis (MSS stage 2) or macular detachment (MSS stage 3 or stage 4), or in combination with vitrectomy when holes are present (MSS stage c) [20]. The macular buckling technique requires precise centration of the buckle in the area of interest. However, we can usually assess its position through the microscope through the panoramic viewing system. The i-OCT confirms the position of the buckle under the fovea and the height of indentation (Supplementary Material Video S9 and Figure 10) and enhances areas with holes centered on the buckle both when working under fluid (Figure 11) and under air (Figure 12).

In a few cases, the i-OCT and the panoramic viewing system represented the only tool that we used to locate the macular buckle during implantation, avoiding the use of endoillumination (Figure 10b and Supplementary Material Video S10). A difficult case of persistent FTMH in a high myopic eye was treated with elevation of the macula with an injection of BSS through a 41-gauge needle, with implantation of the macular buckle to treat the macular detachment, with stretching of the edges of the hole through a scraper and ILM flap which was found dislocated from the previous surgery, and fluid air exchange (Supplementary Material Video S11).

### 3.4. Retinal Detachment (RD)

Totals of 30 (11.32%) and 32 (12.08%) eyes were treated for repair RD and proliferative vitreoretinopathy (PVR), respectively. The i-OCT represented a useful tool in RD repair. Indeed, it showed the surgeon the subretinal fluid status regardless of the tamponade used, as well as offering confirmation of the reattachment of the retina in suspect cases, not only in the macular but also in the peripheral area (Supplementary Material Video S12).

**Figure 13:**
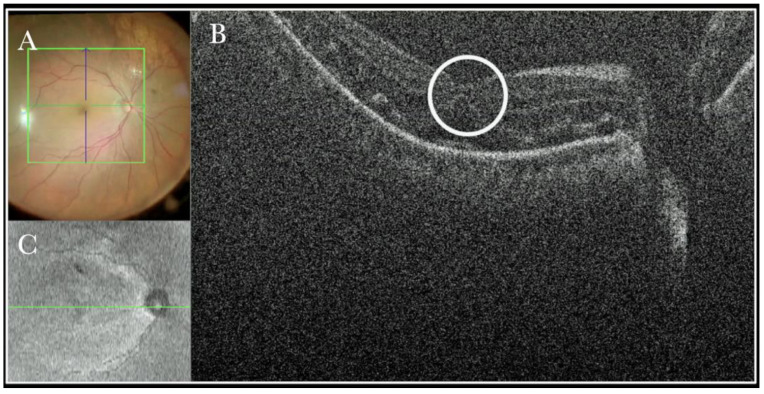
(**A**) Microscope view of the macular area in a case of retinal detachment. The retina is flat under perfluorocarbon liquid (PFCL). The surgeon was considering at this point whether to perform a peeling of the inner limiting membrane (ILM) to prevent the formation of an epiretinal membrane (ERM). (The green box indicates the field-of-view of the OCT scan, the green and blue lines within the green box show the vertical and horizontal scan planes). (**B**) The intraoperative optical coherence tomography (i-OCT) B horizontal scan shows the thinning of the fovea and indicates the surgeon had a high risk of inducing a full-thickness macular hole (FTMH) if performing the ILM peeling. (White borders of circle) a decision not to peel was taken after looking at the i-OCT scan. (**C**) En-face view of the macular area in a case of retinal detachment. (The green line represents the scan plane).

It is debated whether the ILM should be peeled in RD surgery to avoid the secondary formation of ERM [21]. The i-OCT guided the surgeon in deciding when to avoid the ILM peeling in cases of significant thinning of the fovea (Figure 13).

**Figure 14:**
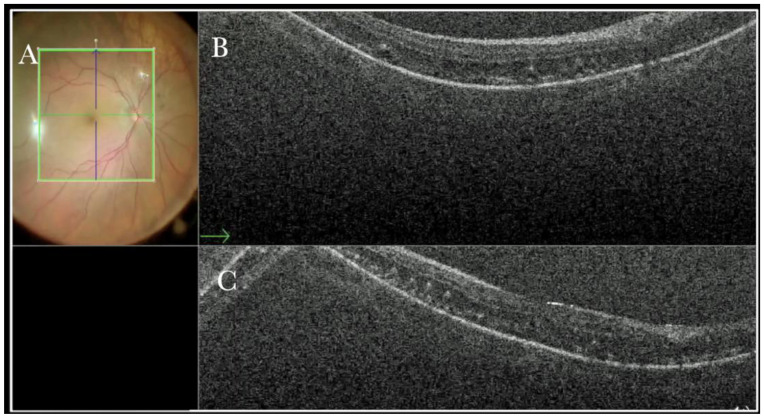
(**A**) Microscope view of the macular area in a case of retinal detachment. The retina is flat under perfluorocarbon liquid (PFCL). (The green box indicates the field-of-view of the OCT scan, the green and blue lines within the green box show the vertical and horizontal scan planes). (**B**) The intraoperative optical coherence tomography (i-OCT) B horizontal (green arrow) scan shows that the retina is flat. The outer layers are slightly edematous and present microfolds. (**C**) i-OCT B vertical scan shows that the retina is flat. The outer layers are slightly edematous and present microfolds.

Furthermore, the i-OCT view helped to study the anatomy of the retina when it appeared fully reattached through the microscope (Figure 14). A series of microfolds were observed in the external layers.

**Figure 15:**
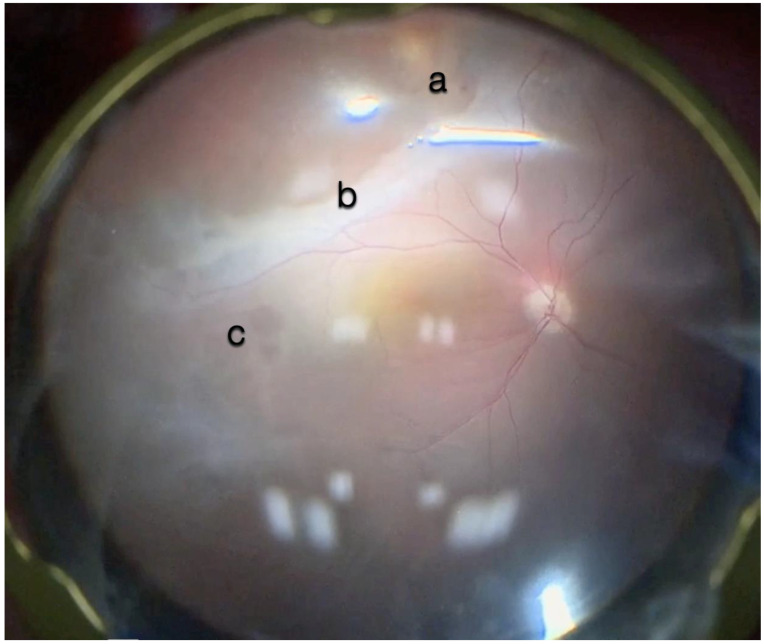
Microscope view of the macular area in a case of retinal detachment associated with retinoschisis. This is the second operation for a relapse of retinal detachment. In the previous surgery, perfluorocarbon liquid (PFCL) was used. The surgeon was considering at this point whether point was (a) a cyst of a hole, (b) schisis or represented subretinal bands or PVR, or (c) indicated the presence of a subretinal PFCL bubble. It is very difficult to discriminate with the microscope view only.

**Figure 16:**
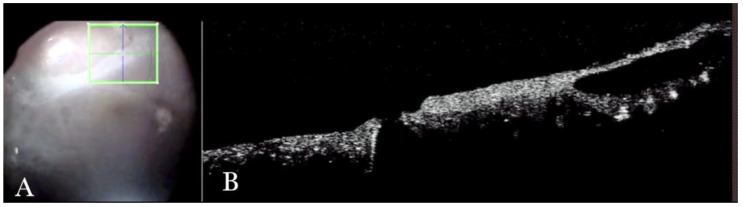
(**A**) Microscope view of point (a) of Figure 15. (The green box indicates the field-of-view of the OCT scan, the green and blue lines within the green box show the vertical and horizontal scan planes). (**B**) Intraoperative optical coherence tomography (i-OCT) can show that, in the area of point (a), there is a hole close to a cyst.

**Figure 17:**
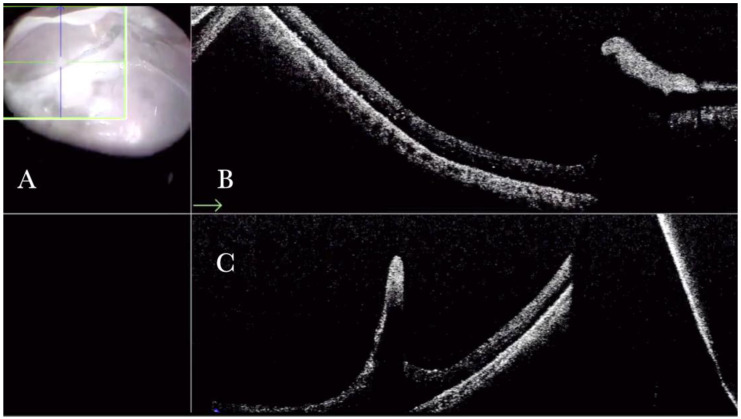
(**A**) Microscope view of point (a) of Figure 15. Microscope view of point (b) of Figure 15. (The green box indicates the field-of-view of the OCT scan, the green and blue lines within the green box show the vertical and horizontal scan planes). (**B**) The intraoperative optical coherence tomography (i-OCT) B horizontal (green) scan shows that, in the area of point (b), the suspect bands are areas of retinoschisis. (**C**) The i-OCT vertical scan shows that, in the area of point (b), the suspect bands are areas of retinoschisis.

**Figure 18:**
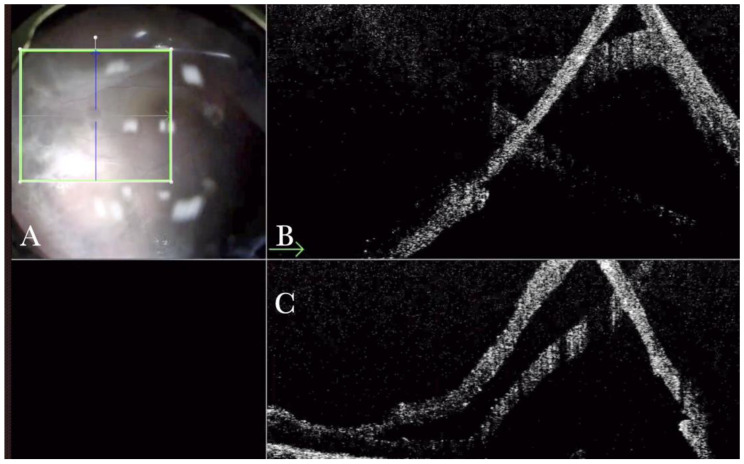
(**A**) Microscope view of point (c) of Figure 15. (The green box indicates the field-of-view of the OCT scan, the green and blue lines within the green box show the vertical and horizontal scan planes). (**B**) The intraoperative optical coherence tomography (i-OCT) horizontal (green arrow) scan shows that, in point (b), the suspect bubbles of perfluorocarbon liquid (PFCL) represent retinoschisis areas. (**C**) The i-OCT vertical scan shows that, in point (b), the suspect bubbles of PFCL represent areas of retinoschisis.

The i-OCT provided a great asset in managing cases of RD associated with retinoschisis (Figure 15).

i-OCT was helpful in discriminating retinal breaks (Figure 16), intraretinal cysts (Figure 16), and areas of schisis (Figure 17), and excluding subretinal PFCL in a relapsed RD secondary to retinoschisis that had previously undergone vitrectomy, PFCL, and silicone oil injection (Figure 18).

**Figure 19:**
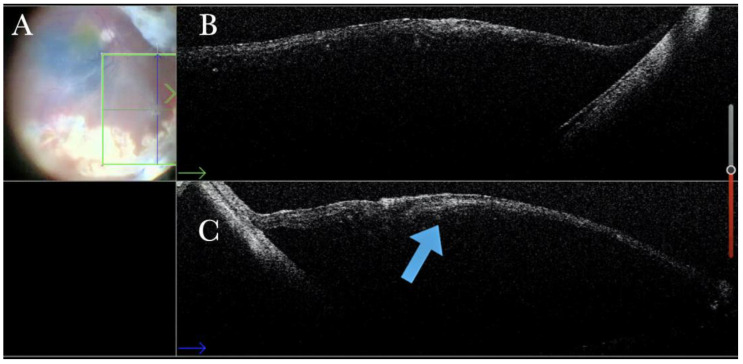
(**A**) Microscope view of a case of retinal detachment with proliferative vitreoretinopathy (PVR). The retina is stained with Doubledyne blue. However, it is not clear where the PVR membranes are located. (The green box indicates the field-of-view of the OCT scan, the green and blue lines within the green box show the vertical and horizontal scan planes). (**B**) The horizontal (green arrow) intraoperative optical coherence tomography (i-OCT) scan shows an area of retina thickening and wrinkling, indicating the presence of an epiretinal membrane (ERM). (**C**) The vertical i-OCT scan (thin blue arrow) shows an area of retina thickening and wrinkling, indicating the presence of an ERM (thick blue arrow).

**Figure 20:**
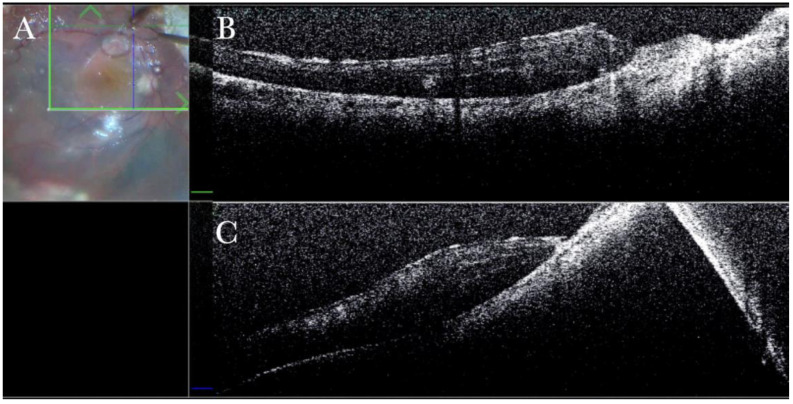
(**A**) Microscope view of the same case of retinal detachment with proliferative vitreoretinopathy (PVR). The retinotomy is analyzed. The surgeon needed to decide whether to peel the epiretinal membrane (ERM) at the edge of the retinotomy and elevate the retinotomy to lower the traction. It is not clear through the microscope how to proceed. (The green box indicates the field-of-view of the OCT scan, the green and blue lines within the green box show the vertical and horizontal scan planes). (**B**) The horizontal (green arrow) intraoperative optical coherence tomography (i-OCT) scan shows the retinotomy, indicating the presence of an ERM and squared edges. (**C**) The vertical (blue arrow) i-OCT scan shows the same area after peeling the ERM, elevating, and reattaching the retinotomy. The retinotomy is now flat and relaxed.

The intraoperative OCT had a significant role in the decision-making process during recurrent RD and PVR cases; for example, detecting minor retinal folds and thickening due to barely visible ERMs leading to a complete peeling (Figure 19) and showing retinal behavior after peeling (Figure 20).

In difficult cases of retina slippage for giant posterior breaks, the i-OCT guided the surgeon in the maneuvers, confirming the correct reattachment of the retina and excluding the presence of subretinal PFCL (Supplementary Material Video S13).

In a case of recurrent retinal detachment with no visible breaks, the i-OCT guided the surgeon in reabsorbing the fluid through a trocar positioned under the retina (Supplementary Material Video S14).

### 3.5. Submacular Surgery to Remove Submacular PFCL

**Figure 21:**
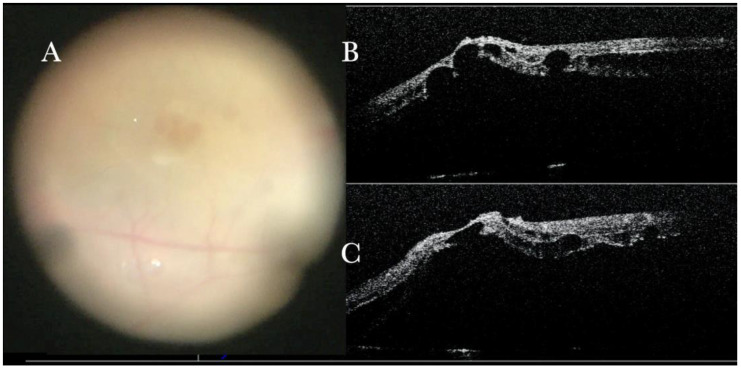
(**A**) Microscope view of the macular area in a case of subfoveal perfluorocarbon liquid (PFCL). (**B**) The intraoperative optical coherence tomography (i-OCT) horizontal scan shows the detached macula with bubbles of PFCL enclosed into the external retina. (**C**) The i-OCT vertical scan shows the detached macula with bubbles of PFCL enclosed in the external retina.

A submacular PFCL bubble is one of the most dreaded complications a VR surgeon can face. A few techniques have been proposed to remove these. The surgeon can choose to detach the macula by injecting a balanced salt solution (BSS), hoping for the bubble to displace away from the fovea. As an alternative, a direct aspiration can be applied in the subretinal space when the bubble does not displace. A third option is to directly aspirate the subfoveal bubble with a backflush needle by creating an FTMH. A case of submacular PFCL is presented below (Figure 21).

A macular detachment was induced by injecting BSS through a 41-gauge needle temporally to the macula. The i-OCT represented a useful tool, showing that the bubble did not displace and instead remained stuck in the external retina (Figure 21), inducing a significant thinning of the retinal tissue. The i-OCT showed that tapping on the retina to displace the bubble did not work. Direct aspiration of the bubble was applied with the 41-gauge needle in the subretinal space, obtaining only partial removal of some of the bubbles. The bigger subfoveal bubble could not be removed. A decision to proceed with direct aspiration with a backflush needle was taken by inducing an FTMH, which was then covered with an ILM flap (Supplementary Video S15).

### 3.6. Submacular Surgery and Autologous Transplantation of Retinal Pigment Epithelium (RPE) and Choroid in Exudative Maculopathy

**Figure 22:**
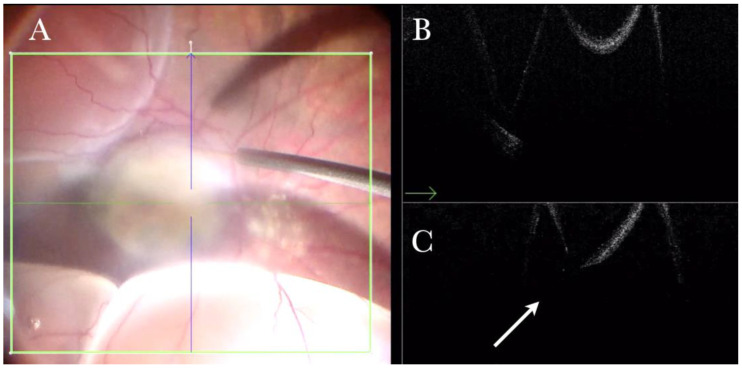
(**A**) Microscope view of the macular area in a case of autologous choroidal transplant. The surgeon is inducing a retinal detachment by injecting a balanced salt solution (BSS) through a 41-gauge needle into the macular area. (The green box indicates the field-of-view of the OCT scan, the green and blue lines within the green box show the vertical and horizontal scan planes). (**B**) The intraoperative optical coherence tomography (i-OCT) horizontal (green arrow) scan shows the detached macula. (**C**) The i-OCT vertical scan shows the detached macula with a full-thickness macular hole (FTMH) (white arrow) induced during the injection of BSS. The FTMH was not visible through the microscope and could be managed during surgery.

**Figure 23:**
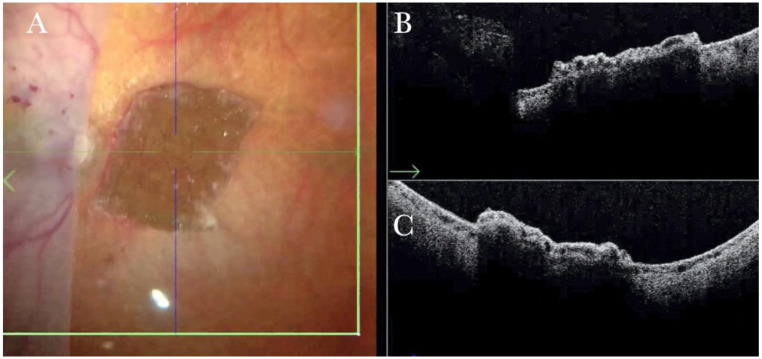
(**A**) Microscope view of the temporal retina that is detached and inverted onto the nasal side. A full-thickness patch of the choroid is visible over the macular area and covered by perfluorocarbon liquid (PFCL). (The green box indicates the field-of-view of the OCT scan, the green and blue lines within the green box show the vertical and horizontal scan planes). (**B**) The intraoperative optical coherence tomography (i-OCT) horizontal (green arrow) scan shows a choroidal patch with no retina on top and covered by PFCL, which is invisible with i-OCT. (**C**) The i-OCT vertical scan shows a choroidal patch with no retina on top and covered by PFCL, which is invisible with i-OCT.

**Figure 24:**
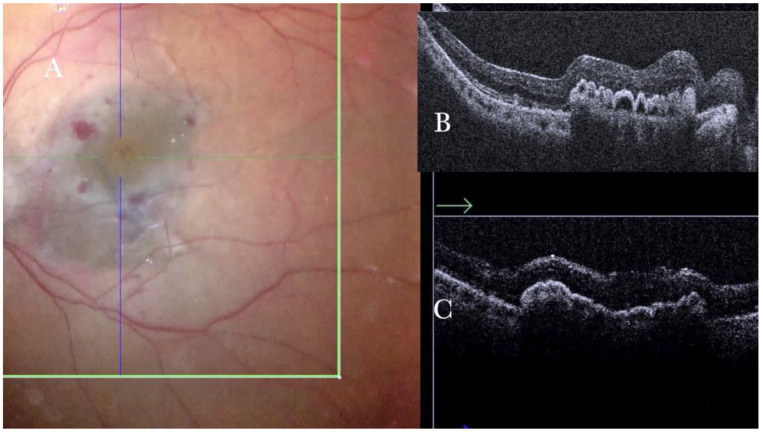
(**A**) Microscope view of the macular area of the same case of autologous choroidal transplant shown in Figure 23. The perfluorocarbon liquid (PFCL) has been moved from the subretinal space to the preretinal space, and the retina has been attached. A full-thickness patch of the choroid is visible in the macular area and covered by the retina. (The green box indicates the field-of-view of the OCT scan, the green and blue lines within the green box show the vertical and horizontal scan planes). (**B**) The intraoperative optical coherence tomography (i-OCT) horizontal (green arrow) scan shows a choroidal patch with the retina on top. The retina appears well-attached. No PFCL bubbles are present under the retina. (**C**) The i-OCT vertical scan shows a choroidal patch with the retina on top. The retina appears well-attached. No PFCL bubbles are present under the retina.

We performed 30 (11.32%) transplantations of the choroid and retinal pigment epithelium for exudative maculopathies. The i-OCT was useful in showing whether an FTMH was iatrogenically induced during the RD (Figure 22), and in displaying the correct position of the patch in the subfoveal area (Figure 23) by excluding the presence of subretinal PFCL (Figure 24).

Furthermore, intraoperative OCT confirmed that the retina was flat after PFCL-silicone oil exchange (Figure 24).

### 3.7. Vitreous Opacities

**Figure 25:**
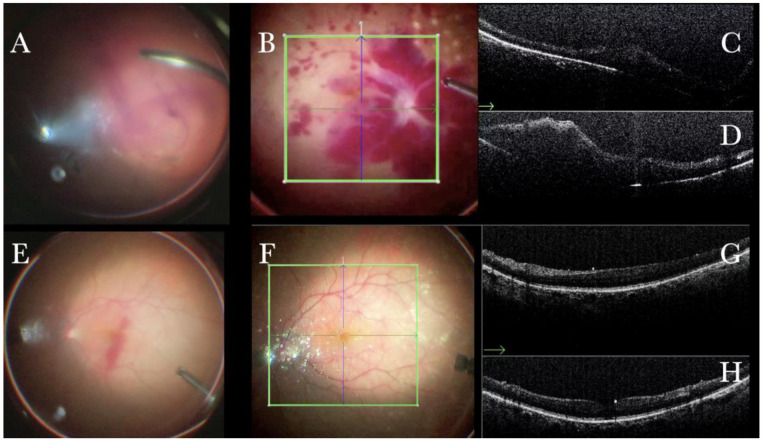
(**A**) Microscope view in a case of vitreous hemorrhage. An OCT was not obtainable prior to surgery. (**B**) Microscope view of the same case after vitrectomy and removal of the vitreous hemorrhage. The macular area is filled with intraretinal hemorrhages. (The green box indicates the field-of-view of the OCT scan, the green and blue lines within the green box show the vertical and horizontal scan planes). (**C**) The intraoperative optical coherence tomography (i-OCT) horizontal (green arrow) scan shows macular thickening but no membranes or holes. (**D**) The i-OCT vertical scan shows macular thickening but no membranes or holes. (**E**) Microscope view in a case of vitreous hemorrhage. An optical coherence tomography was not obtainable prior to surgery. (**F**) Microscope view of the same case after vitrectomy and removal of the vitreous hemorrhage. The macular area seems normal. (The green box indicates the field-of-view of the OCT scan, the green and blue lines within the green box show the vertical and horizontal scan planes). (**G**) The i-OCT horizontal (green arrow) scan shows a normal macular profile. (**H**) The i-OCT vertical scan shows a normal macular profile.

The i-OCT represented a great aid in evaluating, in the real-life setting, the fovea status, which could not be assessed preoperatively due to the shadowing effect of vitreous hemorrhage (Figure 25). Indeed, we could exclusively obtain foveal status details intraoperatively by using the i-OCT.

### 3.8. Effect of Instrumentation on Tissues

**Figure 26:**
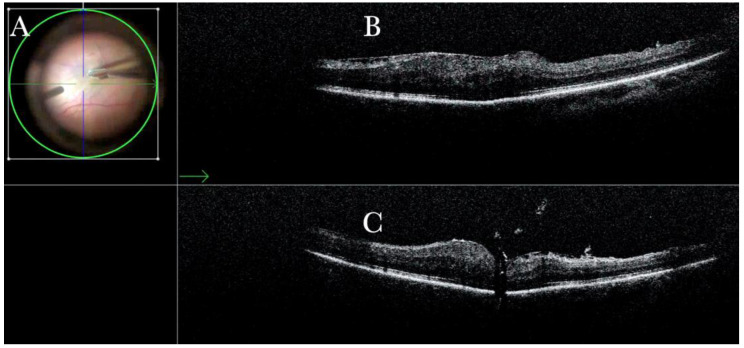
(**A**) Microscope view of the macular area during peeling of the epiretinal membrane (ERM) with toothed forceps. The surgeon has no sense of pressure or touch and counts only on visual feedback to control the maneuvers. (The white box indicates the field-of-view of the OCT scan, the green circle within the white box represents the effective working distance and green and blue lines within the green circle show the vertical and horizontal scan planes). (**B**) The intraoperative optical coherence tomography (i-OCT) horizontal (green arrow) scan shows the difference in the area where the ERM has already been or is not yet peeled, with the effect on the tissue due to the surgical maneuver. (**C**) The i-OCT vertical scan shows the effect on the tissue due to the surgical maneuver of peeling. The tissue is inevitably pushed downward. Possible damage to the retinal layers can be foreseen. The view of the i-OCT’s effect on tissue should guide in improving the technical approach.

**Figure 27:**
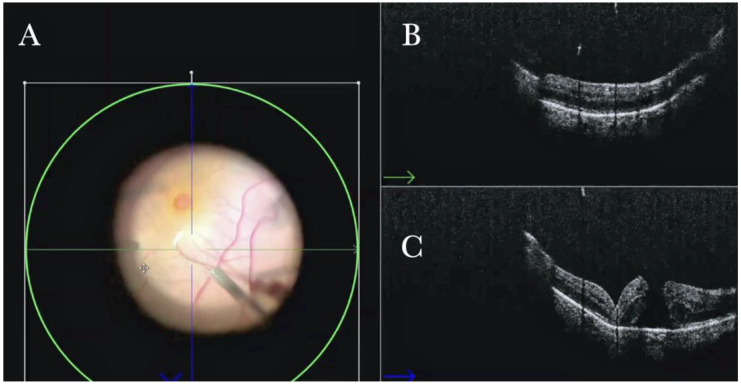
(**A**) Microscope view of the macular area during peeling of the inner limiting membrane (ILM) with a loop. The surgeon has no sense of pressure or touch and counts only on visual feedback to control the maneuvers. (The white box indicates the field-of-view of the OCT scan, the green circle within the white box represents the effective working distance and green and blue lines within the green circle show the vertical and horizontal scan planes). (**B**) The intraoperative optical coherence tomography (i-OCT) horizontal (green arrow) scan shows the difference in the area where the epiretinal membrane (ERM) has already been or is not yet peeled, with the effect on the tissue due to the surgical maneuver. (**C**) The i-OCT vertical (blue arrow) scan shows the effect on the tissue due to the surgical maneuver of peeling. The tissue is inevitably pushed downward. Possible damage to the retinal layers can be foreseen. The view of the i-OCT’s effect on tissue should guide in improving the technical approach.

The i-OCT was a useful tool in assessing the effect of the surgeon’s maneuver on the tissues. The surgeon does not have the feeling of touch and pressure while operating, and, as well, the view through the microscope cannot completely offer the effect of pressure on the tissue. The i-OCT B scan allows us, instead, to observe the different reactions of tissue to a maneuver conducted using different tools (Figure 26 and Figure 27). The view of the effect of the i-OCT on tissue should guide in improving the technical approach.

## 4. Discussion

In this study, we descriptively report the advantages and the feasibility of intraoperative OCT in managing different vitreoretinal diseases in a real-life setting, according to our experience. The results were analyzed per the type of lesion and conditions encountered (Table 3).

### 4.1. Macular Surgery

Macular surgery has specific challenges, such as creating a flap during an ILM peel without trauma to the retina and the determination of where the ILM has been peeled [22]. These issues could be easily overcome by employing i-OCT.

The use of i-OCT in macular diseases, such as ERM, MH, and vitreomacular traction, has been extensively investigated. In these diseases, this technology offers surgeons remarkable imaging of the ERM and tissue planes throughout the surgery [23]. Moreover, it can show subclinical residual membranes and the peeling extension, representing a useful decision-making tool [24].

In 2018, Ehlers et al. assessed the retinal architecture changes which occurred during ERM surgery, finding that i-OCT could identify occult residual membranes in 12% of cases and confirmed complete membrane peeling contrary to surgeon impression in 9% of cases [25]. In our cases, i-OCT allowed us to assess the changes in microarchitectural following membrane peeling in eyes with primary ERM and represented a great asset in examining eyes with severe secondary gliosis, and it confirmed when peeling maneuvers did not induce iatrogenic holes or the presence of residual membranes that required additional membrane peeling.

Furthermore, during surgery, the surgeon’s microscope visualization can be limited by intraretinal hemorrhages, edema, and light reflexes. The view granted by i-OCT is minimally affected by these conditions, allowing us to identify holes’ locations when present, despite the confounding details of the microscope view.

The advantages of intraoperative OCT for FTMHs have been published and reported by many authors [13,24,26,27,28,29,30]. Most of these studies analyzed the geometrical changes in the MHs and the outer retina, and the completeness of peeling, preventing the residual ILM from being left in place [24,27].

While the completeness of peeling can be checked with staining, the i-OCT was helpful in evaluating whether the ILM flap was in place regardless of the tamponade used. The flap was in fact visible even under air tamponade. Maneuvers such as mobilizing the edges of the hole with blunt instruments (scraper, loops), aspirating the fluid into the hole while injecting either air or silicone oil, and stuffing the hole with different tissues (autologous retina or amniotic membrane) are often employed [31,32,33,34,35,36,37].

In our study, the i-OCT allowed us to determine which was the most efficient maneuver to determine the hole closure, guiding us toward the most appropriate surgical strategy. The i-OCT offered a view of the effects of pressure due to the instrumentations and surgical maneuvers on the retina.

### 4.2. High Myopia and MTM

In high myopic eyes, the surgical microscope view cannot offer details of the microstructural changes in the retina, and surgical maneuvers such as ERMs and ILM peeling, as well as ILM flap creations, are often challenging [38,39]. One of the strengths of i-OCT was to enhance the visualization of these transparent tissues and guide our surgery.

MTM entails a wide spectrum of clinical presentations that may affect up to 30% of eyes with pathologic myopia with and without posterior staphyloma [40].

Due to our experience in the macular buckling technique [20,40], we investigated the role of i-OCT in the evaluation of specific surgical steps. The technology was helpful in evaluating the buckle location and offering a precise view of the macular holes’ positions and the height of indentation under any tamponade. Only in selected cases, we implanted the buckle without the use of fiber optics, being guided in the buckle positioning procedure exclusively by the i-OCT.

### 4.3. Retinal Detachment

The PIONEER study showed that, following PFCL application, a variable amount of subclinical persistent submacular fluid remained in nearly all cases. According to their preliminary findings, the amount of subretinal fluid varied significantly, having a prognostic significance for visual outcomes.

In our opinion, the quantification of subretinal fluid under PFCL is only partially meaningful, as that amount of fluid under the macula changes once the PFCL is exchanged with air. Therefore, we used the intraoperative OCT to confirm the subretinal fluid amount at the end of the surgery regardless of the ocular tamponade that was used.

One of the strengths of i-OCT was the ability to differentiate between schisis and RD, retinal breaks and intraretinal cysts, and areas of schisis versus subretinal PFCL.

In recurrent RD and in PVR, the retina is stiff, and the decision making is often challenging. Indeed, when the PCFL is injected, the retinal status is often not clear according to the microscope surgeon’s view. To further guide surgeons in either performing retinotomy or further peeling, the i-OCT could be employed. Indeed, it clearly showed us the retinal behavior in traction-releasing surgical decisions, especially in PVR surgery, where it detected minor retinal folds due to barely visible ERMs leading to a more extensive peeling.

### 4.4. Submacular Surgery and Autologous Transplantation of RPE and Choroid in Exudative Maculopathy

Different studies have investigated the feasibility of i-OCT in submacular surgery and therapeutic delivery procedures, including gene therapy, prosthesis implantation, and subretinal tissue plasminogen activator injection [41,42,43].

Exudative maculopathies, which are nonresponsive to other treatments, might be managed surgically with the transplantation of choroid and retinal pigment epithelium [44,45,46]. The main steps of surgery are summarized as follows: complete vitrectomy, RD of the temporal and superior retina, peripheral retinotomy, folding the temporal retina on the nasal side, choroidal neovascular membrane (CNV) removal, feeder vessel closure, harvesting of a full thickness patch of choroid and transplantation of the patch into the macular area, retinal reattachment on the patch with PFCL, and PFCL exchange either with silicone oil or air-gas. Preserving the macular area and avoiding iatrogenic MHs and residual subretinal PCFL is mandatory for surgical success.

The i-OCT guided the surgeon in evaluating the macular status in real time, and in discriminating between iatrogenic and primary holes. Furthermore, at the end of the surgery, we could assess the presence of subretinal PFCL bubbles, the correct position of the patch, and the retinal status (flat vs. not flat).

### 4.5. Beyond the Visibility

i-OCT images of tissue and tissue-instrument interactions provide additional information, especially in cases of a challenging microscope view. Therefore, combining the enface color view of the microscope with the real-time B-scan, a significant amount of information was retrieved. Supplemental Video S16 shows the versatility of the i-OCT in offering different types of scan lengths, orientation magnification, and shapes in a real-life setting.

Despite being useful in several vitreoretinal diseases, this new technology has some limitations, and the balance among benefits, costs, efforts, human resources involvement, and learning curve should always be considered. Moreover, the absolute shadowing produced by the metallic surgical instruments might restrict the visualization of underlying tissue structures and instrument–tissue interactions [7,13].

Nonetheless, regarding the workflow, the i-OCT did not significantly slow the surgery as it could be easily activated either by a trained technician, able to follow the surgeons during the whole surgery or recording the most significant steps, or directly by the surgeon at any point during surgery via a footswitch or 27” touchscreen HD monitor. Finally, the foot pedal could be fully programmed, allowing the surgeon to change any parameter of the i-OCT, including scanning the area of interest in the x and y-axis, activating the auto sharpen, focus, and contrast, changing the scan length and direction, and selecting among cross, radial, or parallel scans. In real-life experience, the time taken to check the i-OCT becomes, instead, time saved instead of time spent wondering about what we see through the microscope, instead being able to go straight to the next maneuver.

## 5. Conclusions

The i-OCT is a helpful surgical tool in ophthalmic surgery as it provides surgeons with real-time feedback on tissue anatomy, thereby guiding the decision-making process. Moreover, it provides additional information on the microarchitectural changes after instrument–tissue interactions, further guiding procedures when necessary and reducing possible unneeded surgical maneuvers.

Despite the advantages, several limitations have been found across this analysis. First, the processing time for acquiring and analyzing raster scans is strictly related to the surgeon’s and technician’s learning curve. Second, some artifacts, more common for peripheral scans, can lead to misalignment and undocumented areas in the B-scans and 3D reconstructions. Third, we exclusively adopted the EnFocus (Leica/Bioptigen, Durham, NC, USA) as the i-OCT. Finally, we “descriptively” report our experience using the intraoperative OCT, as no control group was enrolled, and no statistical inference could have been made. Therefore, further research evaluating the efficacy and reliability of i-OCT according to confounding factors, such as technician and surgeon learning curves, should be considered. Nonetheless, according to our experience and after a few years of routine usage, we might conclude that i-OCT has become a useful tool for improving the quality of surgery.

## Figures and Tables

**Table 1:** Patients’ demographics.

Features	Number (%)
Sex:	265
Male	131 (49.43%)
Female	134 (50.57%)
Age (years); (mean-range)	58 (10–81)

**Table 2:** Diagnosis features.

Diagnosis	Number, n = 265 Eyes	Sex, Number (%)	Age (Mean-Range)
Myopic Tractional Maculopathy	60 (22.64%)	Male: 18 (6.79%) Female: 42 (15.84%)	58 (39–72)
Epiretinal Membrane	52 (19.62%)	Male: 27 (10.19%) Female: 25 (9.43%)	63 (58–65)
Macular Hole	40 (15.09%)	Male: 25 (9.44%) Female: 15 (5.66%)	68 (12–75)
Retinal Detachment	30 (11.32%)	Male: 18 (6.79%) Female: 12 (4.52%)	58 (37–64)
Proliferative Vitreoretinopathy	32 (12.08%)	Male: 20 (7.55%) Female: 12 (4.53%)	61 (10–68)
Choroidal Transplant	30 (11.32%)	Male: 11 (4.15%) Female: 19 (7.17%)	75 (57–87)
Vitreous Opacities	20 (7.55%)	Male: 12 (4.53%) Female 8 (3%)	71 (57–81)
Optic Disc Pit	1 (0.4%)	Male: 0 (0%) Female: 1 (0.4%)	10

Abbreviations: Number: n.

**Table 3:** Advantages of intraoperative optical coherence tomography (OCT) in managing different vitreoretinal diseases in a real-life setting.

Disease	Advantages of Intraoperative OCT
ERM/ILM	Real-Time Visualization of Surgical maneuvers Evaluation of possible iatrogenic retinal hole
FTMH	Evaluation of the ILM position over the MH regardless of the tamponade Evaluation of the efficacy of the adopted technique Evaluation of ART graft position Evaluation of the presence of ILM flap in high myopic eyes where neither pigmentation nor contrast is present Guiding the decision of “Peel Vs. No peel”
Macular buckle	Evaluation of the buckle position under the MH and fovea
Retinal detachment	Confirmation of retinal status regardless of the tamponade Discrimination between schisis and detachment Discrimination between holes and cysts Evaluation of subretinal PFCL Identification of immature PVR membranes Evaluation of residual traction at the edge of a retinotomy
Media opacities	Intraoperative diagnosis of retinal diseases when the preoperative OCT is not possible due to the opacity of optic media
Any surgery	Real-Time Visualization of Surgical maneuvers

Abbreviations: OCT: optical coherence tomography, ERM: epiretinal membrane, ILM: inner limiting membrane, FMTH: full-thickness macular hole, ART: autologous retina transplant, MH: macular hole, PFCL: perfluorocarbon liquid, PVR: proliferative vitreoretinopathy.

## Data Availability

The data presented in this study are available on request from the corresponding author.

## Supplementary Materials

The following supporting information can be downloaded at: https://zenodo.org/record/7931495 (accessed on 20 August 2023), Video S1: Epiretinal Membrane (ERM) peeling, Video S2: Gliotic ERM peeling performed by a colleague in training, Video S3: Induction of posterior vitreous detachment, Video S4: Inner limiting membrane peeling with risk of inducing a full-thickness macular hole, Video S5: Macular Buckle implantation, Video S6: Persistent full thickness macular hole, Video S7: Macular hole: Inner Limiting Membrane Visco vs. Perfluorocarbon, Video S8: Inner Limiting Membrane peeling in high myopia, Video S9: Macular buckle implant in a case of macular detachment in a high myopic eye affected by myopic macular traction (MTM), Video S10: Management of a persistent macular hole in a high myopic eye, Video S11: Management of persistent myopic full thickness macular buckle macular detachment with Intraoperative Optical coherence tomography, Video S12: Suspicious peripheral retinal detachment, Video S13: Management of a difficult case of retina slippage in a case of a retinal detachment due to a large posterior irregular tear in a high myopic eye, Video S14: Persistent retinal detachment with no visible retinal holes, Video S15: Submacular Surgery to remove submacular Perfluorocarbon liquid, Video S16: Feasibility of the intraoperative optical coherence tomography.
